# Tannic Acid-Loaded Gellan Gum Hydrogels Reduce In Vitro Chemokine Expression in Oral Cells

**DOI:** 10.3390/ijms26125578

**Published:** 2025-06-11

**Authors:** Natália dos Santos Sanches, Atefe Imani, Lei Wang, Otávio Augusto Pacheco Vitória, Hannah Reinert, Layla Panahipour, Francisley Ávila Souza, Idelmo Rangel Garcia Júnior, Reinhard Gruber

**Affiliations:** 1 Department of Oral Biology, University Clinic of Dentistry, Medical University of Vienna, 1090 Vienna, Austria; natalia.s.sanches@unesp.br (N.d.S.S.); n12202452@students.meduniwien.ac.at (A.I.); wanglei20111213@163.com (L.W.); otavio_pacheco00@hotmail.com (O.A.P.V.); hanna.reinert@outlook.com (H.R.); layla.panahipour@meduniwien.ac.at (L.P.); 2 Department of Diagnosis and Surgery, Araçatuba Dental School of Sao Paulo, Sao Paulo 16015-050, Brazil; francisley.avila@unesp.br (F.Á.S.); idelmo.rangel@unesp.br (I.R.G.J.); 3 Wenzhou Institute, University of Chinese Academy of Sciences, Wenzhou 325000, China; 4 Department of Diagnosis and Surgery—Periodontics Division, Araçatuba Dental School of Sao Paulo, Sao Paulo 16015-050, Brazil; 5 Austrian Cluster for Tissue Regeneration, 1200 Vienna, Austria; 6 Department of Periodontology, School of Dental Medicine, University of Bern, 3010 Bern, Switzerland

**Keywords:** tannic acid, gellan gum hydrogel, gingival fibroblasts, inflammatory response, periodontitis, peri-implantitis

## Abstract

Tannic acid (TA), a natural polyphenol with antiphlogistic and crosslinking properties, is a versatile component of hydrogel that can be delivered to inflammatory sites in oral applications. However, the impact of TA dampening an inflammatory response in oral cells remains to be shown. We, therefore, established a bioassay where chemokine expression is induced by exposing gingival fibroblasts and HSC2 oral squamous carcinoma cells to IL1β and TNFα. Additionally, gingival fibroblasts were stimulated with saliva and poly I:C HMW to trigger chemokine production. Our findings demonstrate that TA effectively reduced the expression of *CXCL1, CXCL2, CXCL8, and CXCL10*—in gingival fibroblasts and HSC2 cells—without affecting cell viability. This effect was further confirmed by immunoassays for *CXCL8*. Moreover, we observed that TA decreased *ERK, JNK*, and *p65* phosphorylation in gingival fibroblasts and partially inhibited *NF-κβ/p65* nuclear translocation. Notably, TA released from a gellan gum hydrogel retained its ability to suppress chemokine expression in gingival fibroblasts. These in vitro findings provide insights into the anti-inflammatory properties of TA in oral cells and introduce gellan gum hydrogel as a delivery vehicle paving the way for future preclinical research.

## 1. Introduction

Periodontal disease is a pathological condition caused by microbial imbalance, triggering chronic inflammation and oxidative stress. This catabolic environment destroys the gingiva, periodontal ligament, alveolar bone, and other supporting structures [1,2,3,4]. A similar process occurs in peri-implantitis, culminating in inflammatory osteolysis [2,5,6,7]. Periodontitis and peri-implantitis often arise from the earlier, less severe form of mucositis, which can be reversed with proper oral hygiene, thereby reestablishing the soft tissue barrier [8,9]. Recently, single-cell RNA sequencing revealed the role of oral fibroblasts and epithelial cells in driving inflammation in periodontitis [10] and peri-implantitis [11]. Fibroblasts exhibit a unique ability to recruit neutrophils through chemokine ligands such as *CXCL1, CXCL2, CXCL5*, and *CXCL8*, while *CXCL12, CXCL13*, and *CCL19* attract other leukocytes [10]. Similarly, keratinocytes in periodontitis patients show increased expression of *CXCL1, CXCL3, CXCL8, CXCL13*, and *CCL20* [12], suggesting that chemokine expression in gingival fibroblasts and epithelial cells drives chronic periodontitis. Notably, preclinical research has identified *CCL2* as a key chemokine that is significantly upregulated in periodontitis, promoting inflammation and upregulating pro-inflammatory cytokines such as *TNFα* and *IL1β* [13]. Consistently, increasing concentrations of chemokines such as *TNFα, IL1β* and *CXCL8* in the gingival crevicular fluid and peri-implant crevicular fluid are associated with the severity of periodontitis and peri-implantitis [14,15]. In addition, overexpression of reactive oxygen species (ROS) further exacerbates inflammation and tissue destruction, increasing the risk of implant failure or tooth loss [4]. Thus, lowering the expression of chemokines—thereby dampening the influx of neutrophils [15,16] and other inflammatory cells producing ROS, represents a potential strategy to regain control over periodontal and peri-implant tissues—particularly because conventional treatments, including mechanical debridement and antibiotic therapy, often present limitations [1,2,3]. Consequently, there is a growing demand for adjunctive local strategies to complement conventional periodontal and peri-implant therapies, ideally preventing the progression of mucositis into irreversible tissue destruction.

Tannic acid (TA) is a plant-derived polyphenol approved by the U.S. Food and Drug Administration (FDA) for biomedical applications. Its clinical potential is supported by its potential anti-inflammatory, antioxidant, and antibacterial properties [17]. For instance, TA has been proposed as a local strategy to support wound healing in vivo and in vitro [18,19]. Currently, according to Drug Bank DB09372, TA is topically applied for the treatment of cold sores, diaper rash, fever blisters, and poison ivy. In vitro studies offer insights into the cellular mechanisms of TA, showing that TA reduces cytokine expression in BV2 microglial cells [20], prostaglandin synthesis in irradiated HaCat cells [21], in mouse models of ulcers [22], and in bone marrow-derived macrophages [23]. Additionally, TA downregulates the expression of inducible nitric oxide synthase and other inflammatory mediators in chondrocytes via the mitogen-activated protein kinase (MAPK) and nuclear factor-kappa B (*NF-κβ*) pathways [20,23,24,25]. TA also reduces lipopolysaccharide-induced (LPS) activation of the *Erk2, p38*, and *NF-κβ* signaling in RAW 264.7 cells [26] and decreases ROS production in several models [27,28,29,30]. Given these findings, it is not surprising that TA has been used as a coating for implant abutments [9] and that TA-loaded silver alginate microcapsules hydrogel can support microvascularity in periodontitis models [31]. Together, these findings suggest that TA could combat inflammation in periodontitis and peri-implantitis; however, there is still limited information regarding its potential to reduce chemokine expression in oral cells.

To exert its therapeutic benefits, TA requires a delivery system. As a natural crosslinker, TA undergoes non-covalent interactions such as hydrogen bonds, electrostatic forces, and hydrophobic interactions [32]. Therefore, TA can be incorporated [33,34] and enhance the hydrogel’s stability [35]. Gellan gum (GG) is a naturally derived, anionic polysaccharide polymer that is FDA-approved and commonly utilized in the preparation of situ-forming hydrogels, offering significant potential for drug delivery systems, as discussed in various comprehensive reviews [30,31,32,33]. GG offers excellent biocompatibility, low degradation rates, and tunable mechanical properties, making it an ideal candidate for drug delivery systems [28]. Notably, GG is a thermosensitive hydrogel that remains in a liquid, injectable form at room temperature and undergoes gelation upon exposure to body temperature, making it suitable for applications in drug delivery [36,37,38,39], tissue engineering, and wound healing [40,41]. For instance, GG hydrogel has been used to deliver TA to support wound healing [42,43]. Considering periodontitis and peri-implantitis, the question arises of whether TA, when resealed from GG hydrogels maintains its potential capacity to lower forced chemokine expression using in vitro bioassays. Here, we test the ability of TA to reduce the chemokine expression in gingival fibroblasts and the HSC2 oral squamous carcinoma cell line [44] and evaluate the use of GG as an in vitro TA delivery system.

## 2. Results

### 2.1. Screening of TA Concentrations

**Figure 1:**
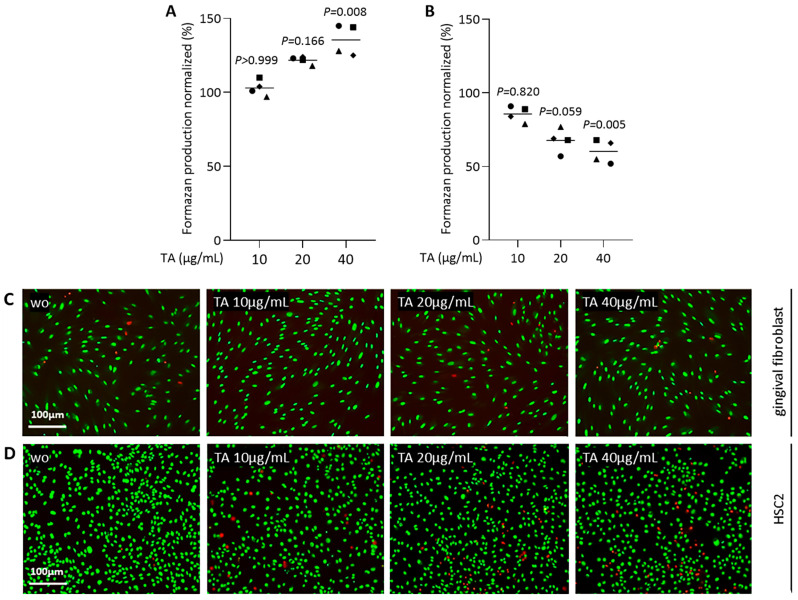
Cell viability assays were performed in gingival fibroblasts (**A**,**C**) and HSC2 cells (**B**,**D**) exposed to serum-free media with a serial dilution of TA (µg/mL) overnight. (**A**,**B**) Viability was assessed by measuring formazan production and expressed as a percentage relative to unstimulated controls. Different symbol shapes represent independent experiments. Statistical analysis was performed using the ratio-paired *t*-tests, and the *p*-values are shown. (**C**,**D**) Representative live/dead staining, with green indicating viable cells and red indicating dead cells. The results suggest cell viability was maintained up to a 20 μg/mL TA concentration for gingival fibroblasts and HSC2 cells. Scale bar 100 µm.

To understand the impact of TA on oral cell viability, we evaluated the ability of gingival fibroblasts and HSC2 cells to convert a tetrazolium substrate into formazan. In gingival fibroblasts, formazan formation increased progressively with higher concentrations of TA (Figure 1A). In contrast, however, HSC2 cells exhibited the opposite trend, with formazan formation decreasing as TA concentration increased (Figure 1B). Cell viability was maintained up to a TA concentration of 20 μg/mL for gingival fibroblasts and HSC2 cells (Figure 1A,B). The predominance of green staining in the life-dead assay further confirmed that cells remain vital at 20 μg/mL TA (Figure 1C,D).

### 2.2. TA Reduced IL1β and TNFα, SLV or Poly I: C HMW-Induced Chemokines in Gingival Fibroblasts

**Figure 2:**
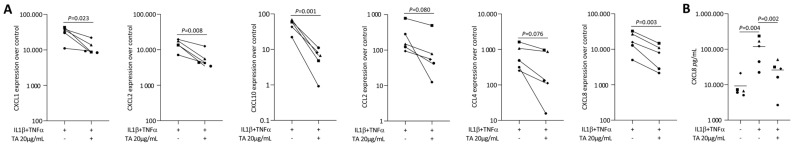
Gingival fibroblasts were exposed to IL1β and TNFα (10 ng/mL) alone and in combination with TA (20 µg/mL) (**A**,**B**). Results were normalized for expression changes for untreated cells. (**A**) Gene expression of chemokines was significantly reduced by 20 µg/mL TA, (**B**) confirmed at the protein level by immunoassay for CXCL8 pg/mL. Different symbol shapes represent independent experiments for PCR and ELISA. Statistical analysis was performed using ratio-paired *t*-tests, and *p*-values are shown.

**Figure 3:**
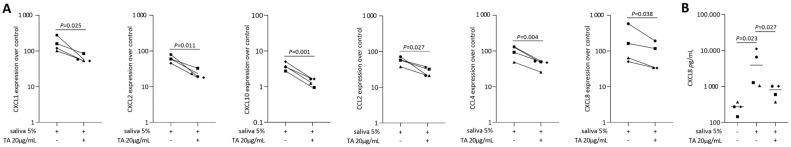
Gingival fibroblasts were exposed to saliva 5% alone and in combination with TA (20 µg/mL) (**A**,**B**). Results were normalized for expression changes for untreated cells. (**A**) Gene expression of chemokines was significantly reduced by 20 µg/mL TA, (**B**) confirmed at the protein level by immunoassay for CXCL8 pg/mL. Different symbol shapes represent independent experiments for PCR and ELISA. Statistical analysis was performed using ratio-paired *t*-tests, and *p*-values are shown.

**Figure 4:**
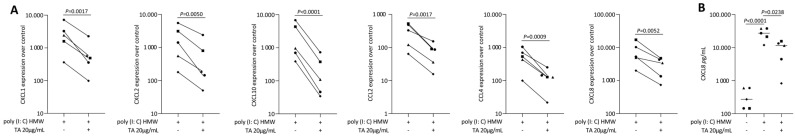
Gingival fibroblasts were exposed to poly (I: C) HMW (100 ng/mL) alone and in combination with TA (20 µg/mL) (**A**,**B**). Results were normalized for expression changes for untreated cells. (**A**) Gene expression of chemokines was significantly reduced by 20 µg/mL TA, (**B**) confirmed at protein level by immunoassay for CXCL8 pg/mL. Different symbol shapes represent independent experiments for PCR and ELISA. Statistical analysis was performed using ratio-paired *t*-tests, and *p*-values are shown.

**Figure 5:**
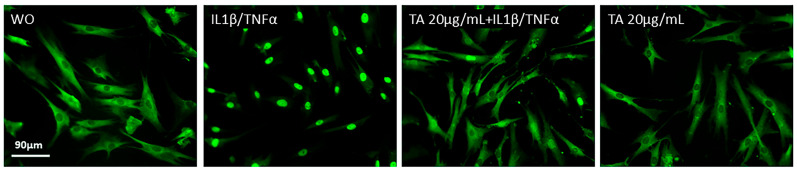
Tannic acid, in combination with IL1β and TNFα (10 ng/mL), partially inhibits the nuclear translocation of p65 in oral fibroblasts. Gingival fibroblasts were untreated (wo; without), exposed to IL1β and TNFα alone, or treated with TA in combination with IL1β and TNFα for 1 h. A faint nuclear p65 labeling was observed in cells treated with 20 µg/mL TA along with IL1β and TNFα, compared to the significant accumulation of p65 in the nucleus in response to IL1β and TNFα alone. The scale bar represents 90 µm.

The anti-inflammatory activity of TA has been reported in various cell types, but its effects on oral cells remain poorly explored [17]. Thus, we hypothesized that TA also exerts anti-inflammatory properties on gingival fibroblasts when exposed to different inflammatory agonists. To test this assumption, we induced an inflammatory response by exposing gingival fibroblasts to IL1β and TNFα, SLV 5%, or poly(I: C) HMW (a TLR3 agonist) [45]. As expected, IL1β and TNFα [38], SLV [38], and poly(I: C) HMW triggered a robust increase in the expression of CXCL1, CXCL2, CXCL8, CXCL10, CCL2, and CCL4 in gingival fibroblasts (Figure 2A, Figure 3A and Figure 4A). We report here that pre-treatment with TA significantly reduced the expression of these chemokines. Gene transcription was supported by an immunoassay showing reduced CXCL8 on the translational level for all the inflammatory agonists (Figure 2B, Figure 3B, and Figure 4B). Consistently with the established role of NFκβ signaling pathways in CXCL8 expression [46,47], exposure to TA in combination with IL1β and TNFα partially inhibits the nuclear translocation of p65 in gingival fibroblasts [17]. Furthermore, TA significantly reduced the expression of MMP1 [48,49], MMP3 [50], and COX2 [17,51,52] (Supplementary Figures S1 and S2) when exposed to IL1β and TNFα or poly(I: C) HMW, highlighting its role in modulating NFκβ activity (Figure 5). These results emphasize the potential of TA to reduce the inflammation and inflammatory damage in gingival fibroblast.

Additionally, since ROS are crucial in inflammation-related tissue damage, the ability of biomaterials to scavenge ROS is important for managing inflammation. In our study, we found that TA treatment significantly reduced mitochondrial ROS levels in gingival fibroblasts exposed to H_2_O_2_ and pro-inflammatory cytokines IL1β and TNFα, bringing them closer to control levels (Supplementary Figure S3A,B) [53,54,55]. Those results underscore a marked decrease in oxidative stress, highlighting TA’s potential as a therapeutic agent to counteract oxidative stress-induced damage in gingival fibroblasts [56].

### 2.3. TA Reduced IL1β and TNFα-Induced Chemokines in HSC2 Cells

**Figure 6:**
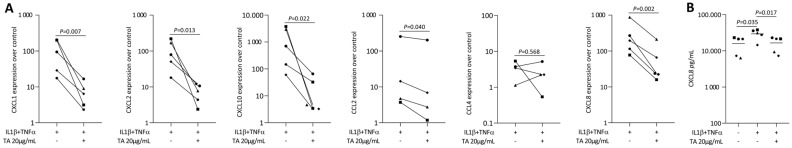
HSC2 cells were exposed to IL1β and TNFα (10 ng/mL) alone and combined with TA (20 µg/mL) (**A**,**B**). Results were normalized for expression changes for untreated cells. (**A**) Gene expression of chemokines was significantly reduced by 20 µg/mL TA, (**B**) confirmed at protein level by immunoassay for CXCL8 pg/mL. Different symbol shapes represent independent experiments for PCR and ELISA. Statistical analysis was performed using ratio-paired *t*-tests, and *p*-values are shown.

To provide direct evidence of TA’s anti-inflammatory activity on oral cells, we conducted additional experiments in HSC2 cells. As we expected, the pre-treatment with TA significantly downregulated the expression of CXCL1, CXCL2, CXCL8, CXCL10, and CCL2 in response to IL1β and TNFα stimulation (Figure 6A). Consistently, the immunoassay demonstrated a decrease in CXCL8 levels at the protein level (Figure 6B). Taken together, these findings highlight the role of TA in reducing inflammation in oral cells.

### 2.4. TA Inhibits the Phosphorylation of ERK, JNK, and p65 in IL1β and TNFα-Induced Gingival Fibroblasts

**Figure 7:**
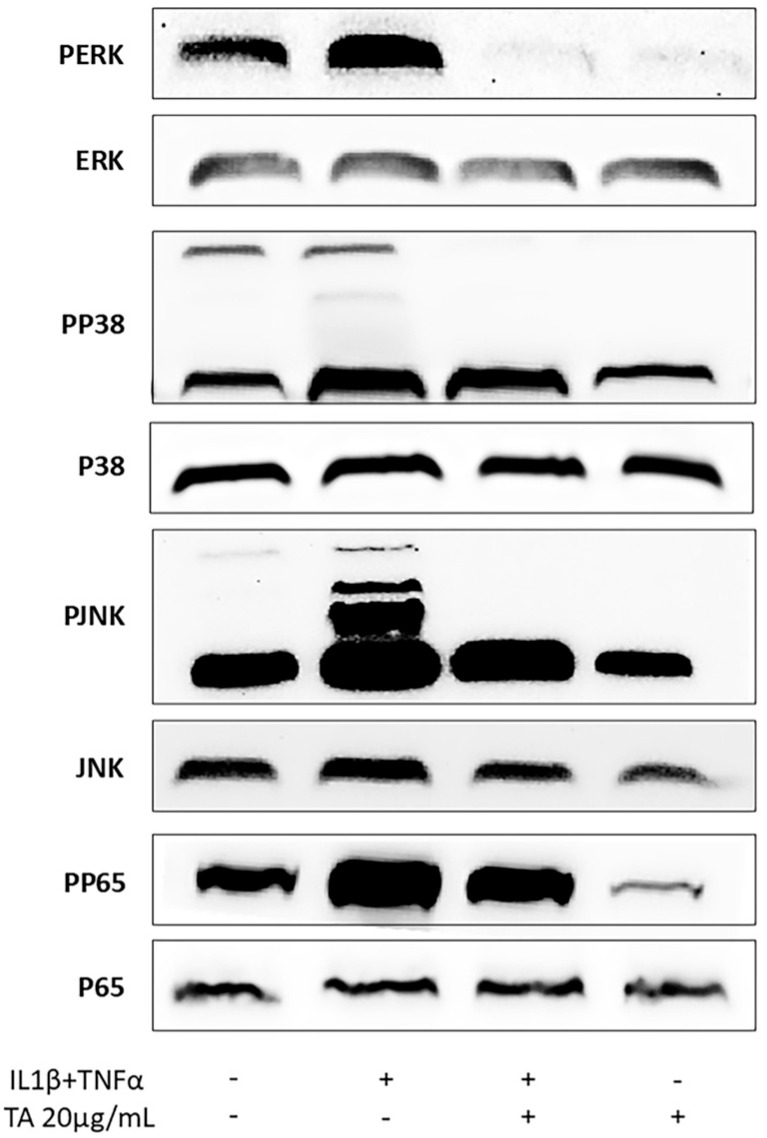
The effect of TA (20 µg/mL) on the phosphorylation of signaling molecules in IL1β and TNFα (10 ng/mL)-stimulated gingival fibroblasts was evaluated. Cell lysates were analyzed by Western blotting to measure both phosphorylated and total levels of extracellular signal-regulated kinase (ERK), c-Jun N-terminal kinase (JNK), p38, and p65.

Next, we further analyzed the MAPK and NFκβ signaling pathways in gingival fibroblasts to investigate the underlying mechanisms of TA’s anti-inflammatory effects. As shown in (Figure 7), IL1β and TNFα significantly enhanced the phosphorylation of ERK, JNK, p38, and p65, while TA alone had no effect. However, TA treatment led to a significant decrease in ERK and JNK phosphorylation [25] with a partial reduction in p38 [27,57] and p65 phosphorylation [27,58]. These findings suggest that TA acts through a broad mechanism, modulating several signaling pathways, including MAPK and NFκβ [17], regulating the phosphorylation of key proteins in the inflammatory response in gingival fibroblast.

### 2.5. Sustained Release of Tannic Acid from Gellan Gum Hydrogel (GGTA)

**Figure 8:**
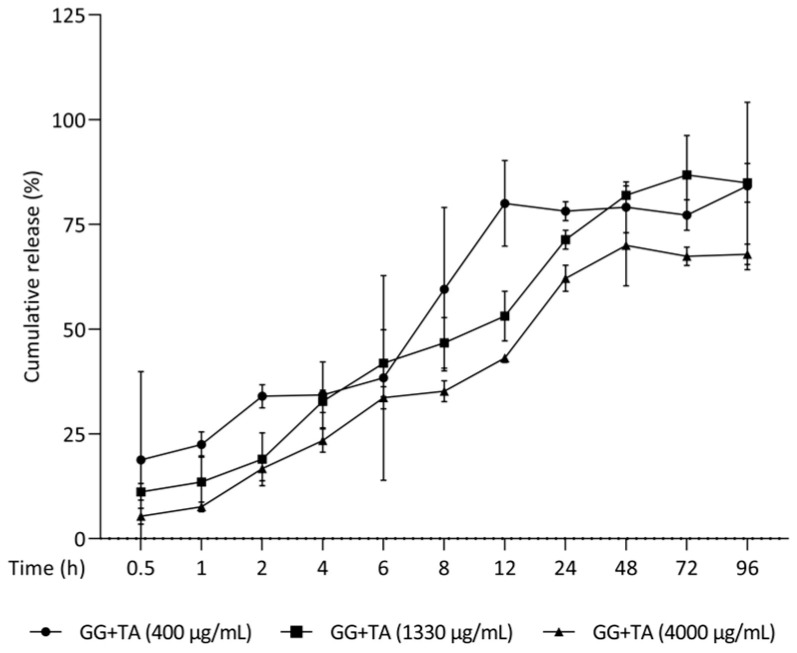
Release the profile of TA at different concentrations of 400, 1330, and 4000 μg/mL from gellan gum (GG) hydrogel in PBS at 37 °C. The long-term sustained release kinetics were monitored from 0.5 to 96 h. Each data point on the release profile represents the mean value ± standard deviation derived from triplicate independent experiments.

Due to its abundant phenolic hydroxyl groups, TA, a plant-derived polyphenol, can form a variety of one-pot physically synthesized hydrogels in combination with different polymers [34]. Previous studies have demonstrated the successful incorporation of TA into hydrogels and hyaluronic acid-based capsules [55]. Building on this evidence, we investigated the potential of GG as a vehicle for topical administration, assessing its ability to modulate TA release in PBS at 37 °C. TA exhibited a sustained release profile, with a gradual, cumulative increase before reaching stability within 72 h (Figure 8), particularly in GG+TA formulations at 1330 μg/mL and 4000 μg/mL. These findings highlight a promising strategy, positioning GG+TA as a potential therapeutic option for the localized treatment of oral disorders in dentistry and oral medicine.

### 2.6. Tannic Acid-Loaded Gellan Gum Reduced IL1β and TNFα or SLV-Induced Chemokines in Gingival Fibroblasts

**Figure 9:**
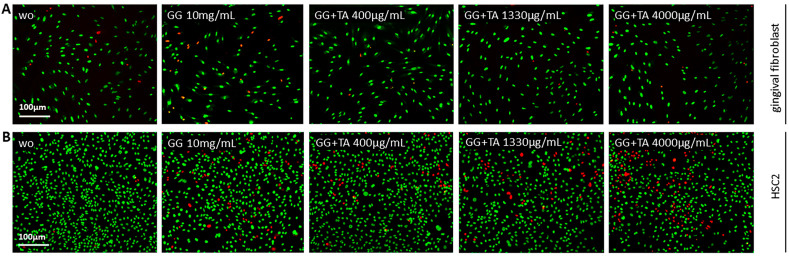
Cell viability assays in gingival fibroblasts (**A**) and HSC2 cells (**B**) exposed to TA at 400, 1330, and 4000 μg/mL from gellan gum hydrogel (GG) after overnight incubation. Representative live/dead staining images show viable cells in green and dead cells in red. “WO” indicates untreated cells, while “GG” represents cells treated with GG (10 mg/mL) without TA. Scale bars represent 100 μm.

**Figure 10:**
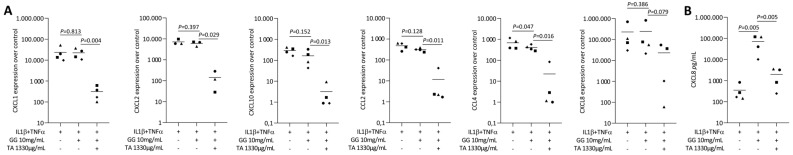
Gingival fibroblasts were exposed to IL1β and TNFα (10 ng/mL) alone and in combination with TA (1330 µg/mL) loaded gellan gum (GGTA) (**A**,**B**). Results were normalized for expression changes for untreated cells. (**A**) Gene expression of chemokines was significantly reduced by GGTA 1330 µg/mL, (**B**) confirmed at the protein level by immunoassay for CXCL8 pg/mL. Different symbol shapes represent independent experiments for PCR and ELISA. Statistical analysis was performed using ratio-paired *t*-tests, and *p*-values are shown.

**Figure 11:**
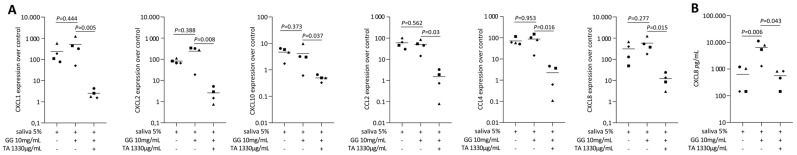
Gingival fibroblasts were exposed to saliva 5% alone and in combination with TA (1330 µg/mL) loaded gellan gum (GGTA) (**A**,**B**). Results were normalized for expression changes for untreated cells. (**A**) Gene expression of chemokines was significantly reduced by GGTA 1330 µg/mL, (**B**) confirmed at the protein level by immunoassay for CXCL8 pg/mL. Different symbol shapes represent independent experiments for PCR and ELISA. Statistical analysis was performed using ratio-paired *t*-tests, and *p*-values are shown.

To further investigate the release of TA-loaded from the GG hydrogel, a coculture bioassay was conducted using gingival fibroblasts and oral HSC2 cells [38]. The live/dead staining assay confirmed the biocompatibility of the TA-loaded GG hydrogel in the gingival fibroblast cellular model (Figure 9A). For the HSC2 oral cells, the results were consistent with previous findings, demonstrating that lower concentrations of TA are safe (Figure 9B). Notably, the presence of GG hydrogel alone appeared to impact cell viability, as indicated by the live/dead staining (Figure 9A,B). Subsequently, we observed that pre-treatment with GG+TA (1330 µg/mL) significantly reduced chemokine expression in gingival fibroblasts stimulated by IL1β, TNFα, and SLV 5% (Figure 10A and Figure 11A). Notably, TA pre-treatment effectively downregulated CXCL1, CXCL2, CXCL10, CCL2, and CCL4 in cells exposed to IL1β and TNFα (Figure 11A), as well as CXCL1, CXCL2, CXCL10, CXCL8, CCL2, and CCL4 in response to SLV 5% (Figure 11A). Gene transcription was supported by an immunoassay showing reduced CXCL8 on the translational level for all the inflammatory agonists (Figure 10B and Figure 11B). Based on the positive results observed, this model supports the potential of TA, either alone or incorporated into GG, as a promising approach for future topical treatments of inflammatory diseases in the oral cavity.

## 3. Discussion

Periodontitis and peri-implantitis are two entities of oral pathologies with a global reach that are linked to chronic inflammation and concomitant catabolic tissue destruction [1]. Apart from prevention, once the disease progresses to mucositis, therapeutic interventions are required to restore tissue health or, at least, to prevent inflammation from advancing to irreversible stages of periodontitis and peri-implantitis. All conventional therapies aim to reduce chronic inflammation’s cause, which is the accumulation of a biofilm and its release of virulence factors provoking inflammation [10,11,12]. However, there is a need for cost-effective and easy-to-use solutions that not only complement conventional therapies but also serve as prophylactic measures. In the case of mucositis, such strategies could help mitigate inflammatory responses, thereby supporting the resolution of the oral disease. Based on this, tannic acid is a possible economical solution, as proposed by an FDA for biomedicine applications [17]; however, its application in dentistry has only started to progress, and its effects on the oral cellular cells remain unclear. Our finding is important because we show that TA is a natural component that is low in toxicity and effectively lowers the forced inflammatory response of gingival fibroblasts and HSC2—oral epithelial cell line cells. Moreover, we have discovered the underlying signaling mechanisms focusing on MAKP and NFkβ signaling.

Relating our findings to previous studies requires acknowledging, apart from the various preclinical models [25,42,43], that in vitro bioassays support the potential of TA to reduce an inflammatory response. For instance, TA could reduce the cytokine expression of IL6, IL1β, and TNFα in BV2 microglial cells [20], human osteoarthritis chondrocytes [24], in mouse models of ulcers [22] and IL1β in BMDMs [23]. Mechanistically, this anti-inflammatory activity is accompanied by TA’s inhibition of MAPK and NFkβ pathway [17,20,23,24]. Building on these consistent findings, our research contributes to this ongoing line of investigation by demonstrating that, at a similar concentration range, TA reduces the expression of CXCL1, CXCL2, CXCL8, CXCL10, and CCL4 without affecting cell viability in primary gingival fibroblasts and HSC2 cells. Furthermore, we revealed that TA decreases the phosphorylation of ERK, JNK, and p65 in gingival fibroblasts. We also observed a significant reduction in p65 nuclear translocation in IL1β+TNFα-exposed gingival fibroblasts, mirroring findings in bone marrow-derived macrophages exposed to LPS [23]. Taking together, supporting previous research on TA released from GG [42,43] and G-sodium alginate hydrogels [59], we demonstrated that TA-loaded hydrogel effectively reduced the induced expression of CXCL1, CXCL2, CXCL8, and CCL2 in gingival fibroblasts, indicating that TA remains bioactive after release from the hydrogel scaffold.

While the clinical implications of our findings remain speculative, the results provide a strong rationale for progressing from in vitro observations to preclinical investigations. Given that chemokine expression by gingival fibroblasts and HSC2 cells plays a central pathological role in periodontitis [10] and peri-implantitis [11], and that TA effectively attenuates this response, our data support its potential for clinical translation—a hypothesis that warrants validation in relevant preclinical models. Notably, TA-gallium ion complexes with polydopamine particles loaded into GG have already been successfully applied in the wound healing model [42]. Similarly, silver alginate microcapsules coated with TA and loaded into GG hydrogel have demonstrated efficacy in periodontitis models [31]—highlighting the synergistic actions of TA, including the reduction in vascular inflammation, ROS scavenging, antibacterial effects, and mitigation of tissue damage. Taking further and considering our findings, we speculate that TA alone or loaded into GG hydrogel could serve as a local anti-inflammatory agent in mouth rinses or toothpaste or, at higher concentrations, be applied to periodontal and peri-implant defects following conventional therapies. Furthermore, it may hold promise as a coating for dental implants to modulate inflammation and promote healing—potentially broadening its applications in the field of dentistry.

Despite the positive outcomes, which offer valuable insight into the potential of TA-loaded delivery systems such as gellan gum hydrogel as topical therapeutic strategies for managing inflammatory conditions in the oral cavity, it is important to acknowledge that our investigation was limited to an in vitro bioassay—a proof-of-principle study focusing on the cellular response of oral cells. In this context, the inflammatory environment was simulated using classical pro-inflammatory cytokines IL1β and TNFα [38], saliva [60], and poly(I: C) HMW, a TLR3 agonist [44]—all of which are mechanistically implicated in driving chemokine expression in gingiva fibroblasts and, to some extent, epithelial cells in vitro. Another limitation of the experimental setup is that the hydrogel was applied only to the edge of the tissue culture plate, covering a limited area. This design allowed a gradient of tannic acid diffusion, with surrounding uncovered cells responding to the released compound, while those directly beneath the hydrogel may have experienced different levels of exposure. Moreover, the intrinsic limitations of in vitro models must be acknowledged, particularly the absence of immune cells, vascular components, and the overall tissue-level complexity that significantly influences drug responses in vivo. To advance these findings, future studies should consider using preclinical models of periodontitis, such as those induced by *Porphyromonas gingivalis* LPS or by ligature placement around the maxillary first molars in rodents. These models effectively mimic chronic inflammation and tissue destruction observed in human periodontal disease. Additionally, omics approaches such as RNA-seq may help uncover broader transcriptomic effects and elucidate specific signaling mechanisms regulated by TA. Further research is needed to translate these findings toward clinical applications in periodontology and implantology, particularly through the local delivery of TA via hydrogel-based systems in periodontal and peri-implant defects. Notably, TA is already commercially available in combination with choline salicylate and cetrimide (Arlak Biotech, Zirakpur, India) and may, in the future, become incorporated into mouth rinses or toothpaste formulations—further reinforcing the clinical relevance of our in vitro findings.

In conclusion, TA—either alone or released from a hydrogel—demonstrates a strong anti-inflammatory potential by modulating chemokine expression and inhibiting MAPK-NFκβ signaling pathways in gingival fibroblasts and HSC2 oral squamous carcinoma cells exposed to IL1β and TNFα. The next steps should include biocompatibility testing, in vivo validation of anti-inflammatory efficacy, and optimization of the hydrogel delivery system for localized and sustained TA release. These findings support the potential of TA-loaded hydrogels for further investigation into preclinical models of chronic inflammation.

## 4. Materials and Methods

### 4.1. Gingival Fibroblasts and Oral Squamous Carcinoma Cells

Primary human gingival fibroblasts were isolated from gingival explants prepared from extracted wisdom teeth of three independent donors, after obtaining informed consent from the patients. The local Ethical Committee approved the protocol (EK Nr. 631/2007). The oral squamous cell carcinoma cell line (HSC2) was obtained from the Health Science Research Resources Bank (Sennan, Japan). All cells were cultured in a humidified atmosphere at 37 °C with 5% CO_2_ in a growth medium consisting of Dulbecco’s Modified Eagle Medium (DMEM; Sigma-Aldrich, St. Louis, MO, USA) with 1% antibiotics (PS; Sigma-Aldrich). Gingival fibroblasts were seeded at a density of 1 × 10^5^ cells/cm^2^, and HSC2 cells were seeded at 2 × 10^5^ cells/cm^2^ into 24-well plates (VWR International, Radnor, PA, USA). The following day cells were pre-treated with tannic acid (TA; CAS: 1401-55-4, Sigma-Aldrich) for 20 min, along with 10 ng/mL IL1β and TNFα (ProSpec-Tany Techno Gene Ltd., Ness-Ziona, Israel), 5% sterile saliva (SLV) [60], or poly(I: C) HMW (InvivoGen, Toulouse, France) in 100 ng/mL [44] as a positive control for inflammation induction. Following overnight incubation under identical culture conditions, RNA was extracted for gene expression analysis, and the supernatant was collected for immunoassay.

### 4.2. Tannic Acid-Loaded Gellan Gum Hydrogel

Tannic acid was dissolved in distilled water with 1% dimethylsulphoxide at 160 mg/mL. The gellan gum (GG, Shanghai Macklin Biochemical Technology Co., Ltd., Shanghai, China) powder was dissolved in distilled water (10 mg/mL) followed by heating at 90 °C for 30 min until reaching transparency. The hydrogel was formulated for this study by mixing TA with GG to reach 400 µg/mL, 1330 µg/mL, and 4000 µg/mL, while GG alone was used as a control. For the delivery assay, the hydrogel was placed perpendicular to the wall of 96-well and 24-well plates seeded with gingival fibroblasts and HSC2 cells, similar to what we have reported and illustrated recently [38].

### 4.3. Release Kinetics of Tannic Acid from Gellan Gum Hydrogel

The in vitro release of TA from a series of drug-loaded hydrogels was studied using a dialysis bag with a molecular weight cut-off of 3500 Da. The dialysis bags were placed in 20 mL PBS in an air shaker at 37 °C with stirring at 100 rpm. At the indicated time, 2 mL of the release medium was collected and replaced with fresh PBS. The kinetics of TA release were determined by multi-mode readers (Synergy HTX, BioTeK, Boston, MA, USA) based on the absorption intensity of TA at 275 nm. The cumulative release of TA was quantified using a calibration curve, which had been previously established through spectrophotometric measurements of TA solutions with known concentrations.

### 4.4. Viability Assay

Viability experiments were conducted on gingival fibroblasts and HSC2 cells, which were treated with serial dilution of TA in a serum-free medium overnight. MTT (3-[4,5-dimethylthiazol-2-yl]-2,5-diphenyltetrazolium bromide; Sigma) was added at a final concentration of 0.5 mg/mL and incubated for 2 h at 37 °C. Formazan crystals were solubilized with dimethyl sulfoxide (Sigma-Aldrich), and optical density was measured at 570 nm. Data were normalized to unstimulated control values. Cell viability was further confirmed using a live/dead colorimetric assay kit, following the manufacturer’s instructions (Enzo Life Sciences, Inc., Lausanne, Switzerland).

### 4.5. Reverse Transcription-Quantitative Real-Time PCR and Immunoassay

For RT-qPCR, after stimulation, total RNA was isolated with the ExtractMe total RNA kit (EUR_x_, Gdańsk, Poland) followed by reverse transcription and polymerase chain reaction (LabQ, Labconsulting, Vienna, Austria) on a CFX Connect Real-Time PCR Detection System (Bio-Rad Laboratories, Hercules, CA, USA). The mRNA levels were calculated by normalizing to the housekeeping gene GAPDH using the ΔΔCt method. The primer sequences of the target genes are indicated in Table 1. For the immunoassay, the human CXCL8/IL8 Quantikine ELISA kit was used (R&D Systems, Minneapolis, MN, USA).

### 4.6. Immunofluorescence Analysis

Gingival fibroblasts were seeded onto Millicell EZ slides (Merck KGaA, Darmstadt, Germany) at 0.5 × 10^5^ cells/cm^2^ densities. Serum-starved cells were pre-treated with 20 μg/mL TA for 20 min, followed by exposure to 10 ng/mL IL1β and TNFα for 1 h, using a serum-free medium as a control. Following treatment, the cells were fixed with 4% paraformaldehyde, blocked using 1% bovine serum albumin (Sigma-Aldrich), and permeabilized with 0.3% Triton X-100 (Sigma-Aldrich). The primary antibody NFκβ P65 (Cell Signaling Technology, Cambridge, UK) was added and incubated overnight at 4 °C. Detection was performed using Alexa 488 secondary antibody (CS-4412, Cell Signaling Technology). Fluorescent images were obtained using a microscope with a DAPI-FITC dual excitation filter (Echo Revolve Fluorescence Microscope, San Diego, CA, USA).

### 4.7. Western Blot

Gingival fibroblasts were serum-starved, pre-treated with 20 µg/mL TA overnight, and exposed to 10 ng/mL IL1β and TNFα for 1 h. Extracts containing SDS buffer with protease and phosphatase inhibitors (cOmplete ULTRA Tablets and PhosSTOP; Roche, Mannheim, Germany) were separated by SDS–PAGE and transferred onto PVDF membranes (Roche Diagnostics, Mannheim, Germany). Membranes were blocked, and the binding of the primary antibody phosphor- ERK and ERK (SCBT; #7383, #81459), phosphor-p38 and p38 (Santa Cruz Biotechnology, SCBT; #4511, #535), phosphor-JNK and JNK (SCBT; #6254, #7345), and phosphor-NFκβ-p65 and NFκβ-p65 (Cell Signaling Technology; #3033, #8242) were detected with the appropriate secondary antibody labeled with HRP (CS-7074; Cell Signaling Technology, Cambridge, UK). After exposure to the clear western ECL substrate, chemiluminescence signals were visualized with a ChemiDoc imaging system. For densitometric analysis of blots, the images were analyzed using Image Lab software 3.0.1 (all Bio-Rad Laboratories, Inc., Hercules, CA, USA).

### 4.8. Mitochondrial Reactive Oxygen Species Release

Gingival fibroblast cells were seeded at a density of 1 × 10^5^ cells/cm^2^ in 96-well plates. The following day, the cells were treated according to the standard stimulation protocol with TA and subsequently exposed to 100 μM H_2_O_2_ (Sigma-Aldrich) [27,61] and to 10 ng/mL IL1β and TNFα [27,53,62,63] overnight. Following the manufacturer’s instructions, the release of the mitochondrial reactive oxygen species (ROS) was assessed using MitoROS^TM^ 580 (AAT Bioquest, Inc., Sunnyvale, CA, USA).

### 4.9. Statistical Analysis

All experiments were conducted at least three times, with each data point representing an independent experiment. Statistical analysis was performed using a ratio-paired *t*-test for single comparisons. All analyses were carried out using Prism V9 (GraphPad Software, La Jolla, CA, USA), with significance set at *p* < 0.05.

## Figures and Tables

**Table 1:** Human primer sequence.

Gene	Forward (5′ → 3′)	Reverse (3′ → 5′)
*hCXCL1*	TCCTGCATCCCCCATAGTTA	CTTCAGGAACAGCCACCAGT
*hCXCL2*	CCCATGGTTAAGAAAATCATCG	CTTCAGGAACAGCCACCAAT
*hCXCL8*	AACTTCTCCACAACCCTCTG	CTTCAGGAACAGCCACCAAT
*hCXCL10*	TGCCATTCTGATTTGCTGCC	TTGGCAGCCTTCCTGATTTC
*hCCL2*	AGAATCACCAGCAGCAAGTGTC	TCCTGAACCCACTTCTGCTTG
*hCCL4*	AATCACCAGCAGCAAGTGTC	TTGGGTTGTGGAGTGAGTGT
*hGAPDH*	TGCACCACCAACTGCTTAGC	GGCATGGACTGTGGTCATGAG

## Data Availability

The original data from this study are provided in the article and the Supplementary Materials section. For additional information, please contact the corresponding author.

## Supplementary Materials

The supporting information can be downloaded at: https://www.mdpi.com/article/10.3390/ijms26125578/s1.
